# Supplementary material for: Are carbon emissions trading and green financial instruments synergistic? -Comprehensive quantitative research based on content analysis
Source: PLoS One. 2024 Mar 7;19(3):e0298601. doi: 10.1371/journal.pone.0298601 (PMC10919601; doi:10.1371/journal.pone.0298601)
Supplement: S1 Data — (ZIP) [file pone.0298601.s001.zip › Data/地级市Z_F工作报告.zip!/╜¡╦╒/─╧═¿╩╨/└┤╫╘╣1⁄2╓┌║┼í╛┬φ┐╦╩2╛▌═°í┐_2013.docx]

各位代表：

　　现在，我代表市人民政府向大会作工作报告，请予审议，并请市政协委员和其他列席人员提出意见。

　　一、2012年工作回顾

　　过去的一年，面对复杂严峻的经济形势，我们在省委、省政府和市委的正确领导下，深入贯彻落实科学发展观，紧紧围绕“两个率先”的目标和“落实‘八项工程’、实现‘八个领先’”的要求，坚持稳中求进，奋力攻坚克难，较好完成了全年目标任务。地区生产总值4558.7亿元，增长11.8%；财政总收入1055.9亿元，地方公共财政预算收入419.7亿元、增长12.3%；固定资产投资2886.5亿元，增长21.4%；社会消费品零售总额1708.7亿元，增长15.5%；外贸进出口总额263.3亿美元，增长1.9%；城镇居民人均可支配收入28292元，农村居民人均纯收入13231元，分别增长12.7%和12.8%；居民消费价格指数涨幅低于省控水平；城镇登记失业率2.48%；全社会研发投入占GDP比重2.2%；基本完成省下达节能减排目标任务。

　　（一）经济发展稳中有进。全力以赴稳增长，千方百计顶住下行压力，经济实力稳步提升。创新政策稳增长。根据经济形势变化，果断采取应对举措，制定进一步保持经济平稳较快发展的7条政策措施和全力稳增长调结构促转型等重点工作意见，实施支持新兴产业和服务业发展、鼓励科技创新、稳定外贸、提升开放型经济发展水平、加快市级中心镇建设等政策措施，把握了经济工作主动权。扩大需求稳增长。组织总投资近2000亿元的400多个项目集中开工。沪通铁路工程可研和广汇能源物流等一批重大项目获批，恒力纺织新材料产业园、三一重工如东工业园等一批基地型产业项目落户、开工，150个市级重大项目完成投资超过800亿元。赴欧美、日本和台湾、上海、武汉等地开展专题招商，注册外资实际到账22亿美元，新增私营企业注册资本986亿元。落实扩大消费政策，加强城市商圈和特色商业街、市场建设，城乡消费不断扩大。优化服务稳增长。全力化解要素瓶颈，各类债务融资总额超过150亿元，中小企业私募债实现零的突破，“区域集优”债务融资取得实质性进展，存贷比提高近2个百分点。加强中小企业服务体系建设，市本级整合设立产业发展资金5.4亿元，全市减轻企业负担超过23亿元。通过努力，主要指标增幅高于全省平均水平、在沿江八市保持领先，规模以上工业产值突破1万亿元。

　　（二）创新转型步伐加快。坚定不移抓创新、调结构，转型升级步伐加快。产业结构加速优化。六大主导产业产值占规模以上工业比重达到90%，家纺产业成为全国区域工业转型升级试点示范产业集群。新兴产业产值接近2600亿元，新培育形成海工、新材料等市级以上特色产业基地13家，10条特色产业链建设有力推进。服务业增加值占GDP比重达到40%。物流、旅游等产业快速发展，农副产品物流中心一期投入运营，濠河景区成为国家5A级景区。建筑业施工产值达到3800亿元。粮食生产实现“九连增”，设施农业面积占比达到14.6%。创新体系加速完善。成为国家创新型试点城市、知识产权示范市，获批创建全国质量强市示范市，新增2家国家创新型企业、2个全国知名品牌创建示范区。全社会研发投入突破100亿元，高新技术产业产值占规模以上工业比重达到39.7%。国家级南通高新区进入审批程序，省级海安、如皋高新区获批，新增省级企业工程技术研究中心和技术中心45家。新增创投和股权投资机构22家。高端人才加速集聚。地方公共财政预算收入的3%用于科技人才发展，“312”产业人才发展行动计划全面实施，新引进国家“千人计划”人才15名、省级人才计划资助对象38名。

　　（三）沿海开发全面提速。把沿海开发作为稳增长、促转型的新空间，着力推进港、产、城融合发展，沿海开发进入加速产出阶段。规划体系不断完善。沿海区域布局总体规划，功能分区和控制性详规，港口、产业、滨海城市规划全面推进，市域高速路网、铁路网、轨道交通网、快速路网、内河航道网等规划全面完成。基础设施快速推进。通洋高速、临海高等级公路建设加快，通州湾快速通道开工，海启高速正式立项。宁启铁路复线电气化改造深入实施。连申线航道整治完成70%的工程量，通吕运河航道升级工程启动。兴东机场国际候机楼开工。洋口、吕四、东灶港一批港口基础设施投入运行，三夹沙匡围工程扎实推进。全市围垦滩涂7万多亩。载体功能加速提升。南通开发区“5+3”特色园区格局形成。海安开发区晋升国家级开发区，苏通科技产业园、吕四港经济开发区获批省级开发区。南通滨海园区建设全面展开，苏通、锡通科技产业园和市北高新（南通）科技城等跨国、跨江合作园区功能有效提升，中奥苏通生态产业园落户。综合保税区获批，洋口港、吕四港国家一类口岸开放申报进入国务院审批程序，家纺产业集聚区海关监管点投入运营。

　　（四）改革开放深入推进。坚定不移以开放促改革、促发展、促创新，发展活力不断增强。开放型经济发展水平实现新提升。新批超3000万美元项目44个、高新技术产业类项目37个。外贸结构进一步优化，国家级外贸转型升级专业型示范基地获批。新批境外投资项目中方协议投资额增长70%。服务外包执行额增长50%以上。争创“江苏民营经济第一大市”迈出新步伐。私营企业数、个体工商户数保持全省第二，民营经济税收、民间投资占全市比重分别提高4.4个、1.4个百分点。新增百亿级特色板块2个，中天科技销售收入超百亿。新增驰名、着名商标34件，省级名牌产品86个。重点领域改革取得新进展。所有制改革扎实推进，国有资源优化整合，沿海开发、城建投资集团成功组建。投融资体制改革不断深化，五大国有资产经营公司融资能力增强，浙商银行南通分行开业。城乡制度设计和创新力度加大，中心城市重点区域建设管理体制调整，沿海前沿区域重点中心镇“区镇一体”管理体制实施。教育、文化、医药卫生体制改革深入推进，事业单位绩效工资改革完成。行政审批制度改革深化，“三集中、三到位”全面推行。

　　（五）城乡面貌明显变化。大力推动城乡统筹发展，城乡面貌加快改善。中心城市和城镇化建设持续升温。中心城市空间战略研究和重点片区规划编制取得重要成果，完成控制性详规、专项规划和城市设计等44项规划。重点区域开发提速。新城区10个项目竣工，商业金融文化集聚区启动建设。观音山新城、市北新城开发建设提速。南部新城形象显着提升。老城区改造提升扎实推进，启动寺街·名人文化区等历史文化保护工程，完成十字街综合改造主体工程。城市功能不断提升。146个重点城建项目完成投资超过200亿元，一批商贸、金融、文化、教育、医疗等功能性项目实现突破。“102030”交通畅通工程取得新成效，5个城市快速路和27个骨干路网项目竣工。初步建成公共自行车服务系统。城市环境展现新貌。市容环境综合整治成效明显。完成城中村改造、老小区整治、城市菜市场升级等项目45个。新建2个综合性公园、5个小游园和一批道路绿化景观带，新增绿地230公顷。城镇建设步伐加快。5个县（市）城新增建成区面积近20平方公里，重点中心镇功能提升。全市城市化率达到58.7%。农村和水利现代化建设扎实推进。农业现代化实现程度达到75.4%。66%的行政村通过村庄环境整治验收。村级集体建设用地试点全面展开，扶持薄弱村集体经济发展成效明显。水利建设投入加大，市区河道整治和农村河道疏浚年度任务全面完成。市区应急水源基本建成。全市城乡居民普遍饮上长江水。城乡生态环境建设不断加强。生态市建设深入推进，国家环保模范城市、国家园林城市通过复核。制定实施加强环保工作的意见，开展“铁腕治污”行动，推进36项环境整治任务，镇村污水处理等三大工程加快实施。引江调水成效明显。秸秆禁烧和综合利用力度加大。连续21年保持耕地占补平衡。新一轮绿色南通建设取得成效，全市新增成片造林面积14万亩。

　　（六）社会建设全面加强。落实加强社会建设构建民生幸福城市和办好民生实事的意见，以改善民生为重点的“十大体系”建设有力推进，地方公共财政预算支出中民生投入比重达到55.6%，10件42项为民办实事项目全面完成。就业和保障水平持续提高。“万人创业”计划深入实施，新增就业8.8万人。城乡基本养老保险、医疗保险覆盖率超过98%，企业退休人员养老金、新农合筹资标准分别提高10%、23.3%，城乡低保标准分别提高10.9%、16.8%，新农合政策范围内住院费用补偿比例超过75%。发放各类救助金8亿多元、惠及30多万群众。市区安置拆迁户1.5万多户，竣工各类保障性住房1795套，低收入家庭住房保障实现应保尽保。文化建设持续加强。精神文明“南通现象”得到弘扬。公共文化服务覆盖面扩大。海安523文化产业主题公园成为国家示范基地，唐闸文化创意产业园开园。一批重大公共文化设施和产业项目快速推进。南通籍健儿在伦敦奥运会、残奥会上勇夺2金2银。社会事业持续发展。区域教育现代化加快推进，省优质幼儿园占比达到69%，城乡义务教育发展实现基本均衡，普通高中实现优质特色发展，职业教育和高等教育发展水平稳步提升。医疗卫生服务体系不断健全，基本药物制度惠民程度提高，基层医疗卫生机构门急诊、住院均次费用分别下降21.8%、17%。优生促进工程全面实施。社会养老服务体系建设加快。社会管理创新持续推进。全国社会管理创新综合试点和重点典型培育工作取得阶段性成效，三级社会管理服务平台建设加速推进。社会稳定、环境风险评估机制全面推行，“四项排查”扎实开展，公众安全感不断提升。基层基础建设进一步加强，矛盾纠纷多元化解、人口服务、新型社区网格化管理等机制完善。食品药品监管扎实有效。安全生产事故总数和死亡人数连续11年双下降。

　　各位代表，一年来，我们以建设人民满意的服务型政府为目标，加快转变职能，自身建设得到加强。服务效能进一步提升。市政务中心投入使用，审批效率提高60%以上。市公共资源交易中心、综合电子监察中心投入运行，综合数据平台初步建成。“12345”政府服务热线平台功能拓展，四级便民服务网络得到完善。运行效率进一步提高。政府工作推进、协调、督查三大体系作用发挥，100项重点工作全面完成。对县（市）区实施重点经济工作综合考核，在政府系统开展“创新奖”评选，促进创新创优。依法行政进一步推进。政府信息公开内容和范围不断扩大，行政权力网上公开透明运行工作有力推进。自觉接受市人大及其常委会的法律监督、工作监督和市政协的民主监督，主动执行市人大及其常委会决议，认真落实市委关于加强政协工作的新要求。全力办好市人大关于区域供水、社会养老服务体系建设的代表议案和市政协关于创新社区管理的建议案，促进了重点工作的落实。全面完成人大代表建议、政协提案办理任务。廉政建设进一步强化。领导干部廉洁自律意识不断增强，行政监察和审计监督力度加大，公务员队伍建设和管理得到加强。

　　各位代表，过去一年各项成绩的取得，是省委、省政府和市委正确领导，市人大、市政协监督支持，全市人民齐心协力、奋力拼搏的结果。我代表市人民政府，向全市人民，向人大代表、政协委员和离退休老同志，向市各民主党派、工商联、各人民团体和社会各界人士，向驻通人民解放军、武警官兵和公安干警以及部省驻通单位，向关心和支持南通发展的海内外朋友，致以崇高的敬意和衷心的感谢！

　　各位代表，我们也清醒认识到工作中还存在不少薄弱环节。增长动力仍然不足，外资、外贸增长乏力，消费拉动不强。经济结构仍需大力优化，服务业和新兴产业比重还不高。创新动力还不强，研发投入占比不高，高层次创新平台、领军型创新人才不足。城乡发展差距仍较明显，城镇化水平有待提高，农村建设仍需加强。社会建设和管理任务仍较艰巨，公共服务与多样化民生需求还不相适应，转型发展中出现的社会矛盾日益增多，一些领域存在道德失范、诚信缺失现象。政府服务效能还需要进一步提升，少数政府工作人员存在推诿扯皮、作风飘浮甚至消极腐败现象。这些都是我们继续努力突破的难点，也是今后工作的重点。

　　二、2013年工作目标

　　今年是全面贯彻落实党的十八大精神的开局之年。综观国际国内形势，世界经济进入深度转型调整期，国内发展方式正在深刻变革。我国重要战略机遇期的内涵和条件发生深刻变化，有利于我们在调整结构中做强实体经济，在转型升级中实现稳定增长，在加快发展中改善民生；江苏沿海开发和长三角一体化发展两大国家战略深入实施，省委、省政府支持南通建设长三角北翼经济中心，有利于我们赢得更多发展支持，拓展更大发展空间；总体宽松向好的宏观政策环境，有利于我们扩大内需，支撑增长；我市具有良好的发展基础和稳增长的宝贵经验，有利于我们提振信心，保持奋发有为的精神状态，把南通的发展不断推向前进。同时，我们既面临经济增长的下行压力、区域竞争的拼抢压力，又面临瓶颈制约加剧带来的攻坚压力、利益格局多元带来的维稳压力。面对机遇与挑战，我们必须解放思想，凝心聚力，以更强的责任意识促发展、增优势，以更强的忧患意识找差距、争赶超，推动各项工作再上新台阶。

　　今年是实施“十二五”规划承前启后的关键一年。我们必须着眼“十二五”后三年发展，围绕“五位一体”总布局，按照率先全面建成更高水平小康社会、率先在江苏江北基本实现现代化和建设长三角北翼经济中心的目标，推动“四化”同步发展，毫不松懈抓好创新驱动、江海联动、经济国际化、城乡发展一体化、民生优先、可持续发展“六个战略着力点”，加快形成新的发展优势。一是坚持优化结构，大力推动经济发展转型。以新兴产业基地和重大载体建设为突破口，加快形成以高新技术产业为导向、先进制造业为支撑、现代服务业为特色、高效农业为基础的现代产业体系，构筑结构合理、技术先进、特色鲜明的经济发展格局。二是坚持创新发展，大力推动发展动力转型。以创新平台支撑和人才引领为突破口，加快形成开放式、网络化、集聚型的区域创新体系，构筑创新主体不断壮大、创新载体加速提升、创新人才充分集聚的内生增长格局。三是坚持城乡统筹，大力推动城乡结构转型。以城镇功能提升和空间形态优化为突破口，加快形成以中心城市为龙头，中等城市、小城市、特色镇为支撑，农村新型社区为基础的城乡架构体系，构筑功能完善、统筹协调的城乡融合发展格局。四是坚持改善民生，大力推动社会发展转型。以收入结构调整和公共产品供给为突破口，加快形成政府主导、覆盖城乡、可持续的基本公共服务体系，构筑成果共享、安定和谐的社会建设格局。五是坚持以人为本，大力推动人的发展转型。以教育文化引领和生态文明提升为突破口，加快形成布局科学合理、素质整体提升、环境宜居宜业的人的全面发展促进体系，构筑生产、生活、生态相和谐的可持续发展格局。六是坚持改革开放，大力推动体制机制转型。以政策设计和管理创新为突破口，加快形成系统完备、科学规范、运行有效的制度体系，构筑多种所有制竞相发展和经济国际化快速提升的开放格局。

　　今年政府工作的总体要求是：深入学习贯彻党的十八大精神，以邓小平理论、“三个代表”重要思想、科学发展观为指导，按照中央、省委省政府和市委一系列重要会议部署，紧紧围绕主题主线，以提高经济增长质量和效益为中心，以改革开放和创新驱动为动力，以保障和改善民生为重点，稳中求进，开拓创新，扎实开局，继续落实“八项工程”，奋力实现“八个领先”，保持经济持续健康发展和社会和谐稳定，开创“两个率先”新局面。

　　今年经济社会发展的预期目标是：地区生产总值增长11%左右；地方公共财政预算收入增长11%以上；固定资产投资增长18%以上；社会消费品零售总额增长15%；外贸进出口总额增长6%左右；城镇居民人均可支配收入、农村居民人均纯收入均增长13%；居民消费价格指数涨幅低于省控目标；城镇登记失业率控制在4%以内；全社会研发投入占GDP比重达到2.3%；万元地区生产总值能耗降幅和主要污染物减排完成省下达任务。

　　三、2013年主要任务

　　实现今年目标，我们将重点抓好十个方面工作：

　　（一）着力推进产业升级，加快提升发展质量。坚持走新型工业化道路，以特色产业基地建设为抓手，主攻经济结构调整，突破重大产业项目，在转型升级中实现规模做大、质量提升。

　　提升发展主导产业。围绕船舶及重装备等六个重点板块，以项目为抓手，推动主导产业向高端化、集聚化、品牌化发展，力争产值超过1万亿元。围绕主导产业提升、支柱产业做强，引进龙头企业，增强配套能力，拉长产业链，提高附加值。推动工业化与信息化深度融合，实施一批技术改造项目，培育20家信息化示范、试点企业，促进制造业层次提升。推动建筑业转型升级，施工产值突破4000亿元。

　　突破发展新兴产业。围绕“3+4”重点领域，加快实施新兴产业发展“310”工程，确保新兴产业产值超过3000亿元。实施新兴产业基地规模化发展行动计划，在海洋工程、新材料、新能源、生物医药、智能装备、节能环保等领域实施一批重点项目，新培育市级以上特色产业基地10家。完善10条特色产业链，突破关键项目、核心技术、配套能力，形成新兴产业竞争新优势。以信息基础设施建设带动“智慧城市”建设，推动信息产业和信息服务业发展。加快推进空港、光电等特色园区规划建设。坚持新型工业化与城镇化互动，在有条件的镇培育新兴产业特色园区。

　　集聚发展现代服务业。围绕“7+3”重点领域，加大推进力度，确保服务业增加值突破2000亿元。实施服务业集聚区提升工程，打造一批集聚型示范区。大力发展现代物流、金融和科技、商务服务等产业，力争生产性服务业增加值占服务业比重超过40%。大力培育旅游休闲、文化健身、家政服务、社会养老等消费热点，积极发展信用、网络消费等新型业态。推动先进制造业与现代服务业融合发展，深化企业主辅分离。推进168个服务业重点项目建设，确保服务业投资超过1300亿元。

　　大力发展现代农业。围绕优质粮油、茧丝绸、水产等十大特色产业，大力推进农业产业化经营，农业现代化实现程度达到78%。加快农业结构调整，做大做强外向农业、品牌农业、观光休闲农业，新增高效设施农（渔）业面积9万亩以上。加强农业园区建设，新建农（渔）业园区、基地项目16个以上。构建集约化、专业化、组织化、市场化相结合的新型农业经营体系，大力发展农村合作经济组织，推进“全托管”式农业服务体系建设，新增市级以上龙头企业35家，农民专业合作组织农户入社率超过85%，提高农业科技推广、标准创建、农机装备和综合服务水平。

　　（二）着力推进创新驱动，加快构建区域特色创新体系。以科技创新引领发展方式转变，推动国家创新型试点城市建设。

　　加快推进技术创新。完善以企业为主体、市场为导向、产学研相结合的技术创新体系，增强产业创新能力。加大“五自”企业培育力度。构建科技型企业、高新技术企业、创新型领军企业梯队发展格局，新增省级以上研发平台超过10家，实现大中型先进制造业企业研发机构全覆盖，确保高新技术产业产值达到4600亿元。加大应用研发力度。开展10项重大产业技术集中攻关，实施20个重大产业化项目，增强企业开发新技术、发展新产品、创建新品牌的能力。加大产学研合作力度。支持企业提前介入知名高校、科研院所原创性科研项目的前期研发，鼓励县（市）、镇与高水平大学、院所开展合作，大力推进产学研协同创新，力争国内外200项重大科研成果在通转化。

　　加快建设创新载体。完善以高新区为龙头、创新核心区为重点、科技园区为基础的创新载体体系，加速集聚创新资源。推进高新区创建提升，力争南通高新区成为国家级高新区，新增省级高新区1-2家。推进创新核心区建设提速，每个县（市）区新建创新载体不少于5万平方米。推进重点科技园区发展提质，强化产业化对接、专业化发展，力争建成3-4家特色产业技术研究院。

　　加快构筑人才高地。完善富有竞争力的引才、用才、留才和服务人才工作体系，放大人才引领优势。深入实施“江海英才”计划和“312”产业人才发展行动计划，引进国家“千人计划”人才20名、各类科技领军人才500名。加强人才创业载体和支持服务体系建设，营造一流人才环境。打造一支懂产业、懂技术、懂人才规律的工作队伍，为人才提供更加专业化、全方位的服务。

　　加快优化创新环境。完善政策扶持体系，整合各类资源，重点支持创新平台加快建设、提升水平。强化科技创新服务平台功能，大力建设关键共性技术研发与公共服务平台、行业技术与产品开发平台。加大知识产权创造、保护和运用力度。完善多元化科技投入机制，加大财政投入力度，大力发展创投和股权投资等机构，力争建成省级科技金融示范区。

　　（三）着力推进江海联动开发，加快打造新的增长极。以优化生产力布局为抓手，放大江海联动开发特色优势，力争沿海前沿区域地区生产总值和固定资产投资均突破1000亿元。

　　统筹开发优化布局。继续深化沿海总体布局、功能分区等规划，制定实施全市产业空间布局指导意见，以规划提升沿海开发科学布局水平。统筹沿江提升与沿海开发。推进“优江拓海”，推动市区等重点区域产业布局优化与梯度转移，不断提升沿江产业集约化、高端化水平，加快建设沿海新兴基础产业基地和海洋产业集聚区。统筹陆域建设与海域开发。积极争取国家陆海统筹发展试点，制定发展海洋经济意见，整体规划、科学利用陆海空间资源，增强对发展的承载力。统筹港、产、城融合开发。完善综合交通“五张网”。开工建设沪通铁路。力争临海高等级公路主体完工，通洋高速、通州湾快速通道累计完成总投资70%以上，海启高速开工建设；兴东机场国际候机楼、汽车客运枢纽东站投入使用。建成连申线航道南通段，推进江海河联运项目。新建、扩建变电站20座。推进10个港口建设项目，确保通州湾起步码头工程开工，南通港吞吐量突破2亿吨。实施沿海前沿区域城镇功能完善和产业体系构建工程，推进30个城镇功能性项目和60个产业项目建设，集聚要素，提升功能。

　　集约开发优化布局。大力发展园区经济，提高产业集中布局水平。推动开发区转型升级。以体制创新、功能配套和升级扩容为重点，打造一批产城融合示范园、特色“区中园”，新增1-2家国家级、省级开发区。推动沿江沿海园区特色发展。推进南通滨海园区形态开发和功能开发，争创国家新兴产业战略合作示范区和海洋经济示范区。支持县（市）临海产业园区做强特色产业，加速提档升级。推动合作园区、特殊功能园区做大做强。促进苏通、锡通科技产业园等合作园区快建设、多产出，中奥苏通生态产业园全面启动建设。加快综合保税区建设，推进重点口岸开放。

　　联盟开发优化布局。推进联盟合作，拓展沿海开发空间。深入接轨上海。积极承接辐射，主动对接和服务长三角一体化发展。加强与长江流域城市合作。面向重庆、武汉等城市，组织开展系列交流对接活动，力争在港口等资源联合开发和物流等产业联动发展上取得突破，促进沿海资源与长江流域共建共享。

　　（四）着力推进中心城市建设，加快增强城市综合竞争力。深入实施“繁荣、提升、整合、扩容、解难、服务”六项举措，打造“江海明珠、灵秀南通”新形象。

　　以规划体系完善引领城市发展。立足城市转型，以高水平规划推动中心城市由带状向“一核三片区”的T型城市组团发展。以新一轮城市总体规划为指导，实现城市规划建设用地控规全覆盖。完善重点区域布局规划，进一步明确各板块要素构成、功能组合和业态分布。加强城市设计，强化对城市空间形态和建筑风格、色调引导，促进城市特色更加鲜明。

　　以重点区域开发完善城市功能。围绕构建“1+5+8”空间布局体系，推动重点区域建设提速、功能完善。加快新城区繁荣。推动商业金融文化集聚区、通沪大道两侧等开发突破。加快观音山新城开发，基本完成起步区建设。加快南部新城建设。以能达商务区、苏通科技产业园为重点，加速形成城市国际化新亮点、新形象。加快市北新城提升。实施一批城市综合体、生态景观、文化体育等功能性项目，完善核心区城市功能。加快通州区与主城区融合。推进组团连接通道建设，促进金沙城区、先锋地区等重点板块功能出新、形象提升。

　　以综合整治改造提升城市品位。完善环境整治、更新改造、科学管理等长效机制，让城市更靓丽。按照“精致、细腻、整洁、有序”的要求，以“八横八纵一环”路网沿线整治为重点，持续改善城市面貌。完善城市管理体制，创新数字化、网格化管理机制，提升城市科学化管理水平。有序推进“四老”更新改造，加快寺街·名人文化区等项目建设，完成15个“城中村”和3个老小区改造项目。

　　以重大工程建设优化城市服务。组织实施305个城建项目，完善城市为民服务体系。推进公共交通建设。加快71个道路项目建设，基本建成市区快速路体系，进一步完善骨干路网和支路网。深入实施“公交优先”战略，提升硬件设施和软件配套水平，让市民出行更方便。推进公共空间打造。启动国家生态园林城市创建工作，加快滨江大桥桥头公园等6个公园和一批小游园建设，整体打造“两河两岸”景观带，完善社区文化、体育等公共设施，为市民创造优美舒适的休闲空间。推进公用事业发展。实施25个公用事业项目，建成崇海水厂，优化城市水电气服务。

　　以做强城区经济支撑城市发展。强化城区各区域二三产业合理布局，推进一批2.5产业、总部经济、楼宇经济、金融集聚区项目，打造一批文化、创意、家居、商贸等特色街区，促进城区经济做大规模、提升层次。

　　（五）着力提升城镇化质量，加快推动城乡发展一体化。把城镇化作为城乡发展一体化的重要抓手，把城乡发展一体化作为解决“三农”问题的根本途径，加快构建新型工农、城乡关系。

　　强化城乡发展一体化支撑。抓住城镇化建设机遇，增强中等城市、小城市、特色镇综合承载能力，力争全市城市化率达到60%。推进各县（市）城市总体规划修编，加快提升中等城市能级。全面落实支持市级中心镇发展的指导意见，加大规划引导、资源整合力度，推动15个左右市级重点中心镇向小城市发展。因地制宜培育一批特色镇，努力形成更多新的增长点。

　　夯实城乡发展一体化基础。深入推进新农村建设，突破城乡统筹发展薄弱环节。加大强农惠农政策力度。财政“三农”投入增长22%，让农民分享更多改革发展成果。提升农村生产生活条件。推进农村干线路网建设和农用桥梁改造。新建高标准农田20万亩。加快水利现代化进程，推进中小河流治理、沿江提水泵站、江海堤防提标等水利工程，完成水利建设投入30亿元以上。继续实施“四位一体”长效管理，深入推进村庄环境整治，力争所有行政村通过验收。巩固区域供水和农村饮水安全工程实施成果。加强村级“四有一责”建设。支持村级集体经济发展，确保50%经济薄弱村集体经营收入超过30万元，以强村带富民。

　　创新城乡发展一体化制度。围绕城乡规划、产业布局、资源配置、基础设施、公共服务、就业社保、生态文明、社会管理“八个一体化”，加强政策设计和制度创新。稳妥实施户籍制度改革，加快农村土地制度创新，继续推动“三集中、三置换”，有序推进农业转移人口市民化。加大土地流转政策扶持力度，提高农民在土地增值收益中的分配比例。加快农村金融制度创新，积极发展小额贷款公司、合作金融、村镇银行等机构。有序实施农村集体经济股份制改革。在有条件的地区推进城乡社会保障并轨。

　　（六）着力推进改革开放，加快经济国际化进程。更好处理政府与市场关系，更大力度抓好改革开放。

　　大力发展开放型经济。全方位推进企业、人才、城市、载体、环境国际化，放大开放型经济优势。提高利用外资水平。培育利用外资特色产业链基地，打造专业化、精细化招商队伍，确保注册外资实际到账25亿美元，引进超亿美元项目18个。推动引资、引技、引智、引业态结合，着力引进区域性或职能型总部。优化对外贸易结构。落实支持外贸发展政策措施，推进科技、品牌、基地建设兴贸，扩大优势产品出口。加快以电子口岸为重点的“大通关”体系建设，推动进、出口协调增长。做强服务外包产业。更大力度建设服务外包园区，壮大研发设计产业，突破金融外包、云端服务等领域，确保服务外包执行额增长30%以上。拓展外经合作深度。支持重点企业到境外设立加工贸易基地、技术研发中心，推动对外承包工程和成套设备出口企业“走出去”。发挥外事、侨务、港澳事务、对台事务在扩大开放中的作用。

　　加快民营经济国际化进程。毫不动摇鼓励支持民营经济发展，促进民营企业做大做强做优。以总量扩张促提升。全面落实国家“新36条”实施细则，积极推动全民创业，在降低融资成本、保障要素配置、优化政策服务上更有力地支持实体经济发展，确保民营经济增加值占GDP比重提高1个百分点。以转型升级促提升。制定支持民营经济转型升级的政策意见，以培大扶强、品牌打造、增创特色带动转型，新增一批百亿级产业板块和营业收入超50亿元企业，争创驰名、着名商标28件，省级名牌产品75个。以融合发展促提升。支持民营企业借助资本市场、国有资本和外来资本加快国际化进程，力争新增上市企业3家。

　　深化重点领域改革。推进财税体制改革，完善税收征管体制。优化国有资产管理体制，增强国有资本运作能力，在资源资产化、资产资本化、资本证券化上实现新突破，促进央企资本、外资、民资融合发展。健全多元化投融资体系，加大金融创新力度。构建现代文化市场体系。加快社会领域改革，推进教育、卫生等体制改革，深化公立医院改革。加强要素市场建设。

　　（七）着力推进文化强市建设，加快文化事业和产业发展。放大江海特色文化优势，推动文化大发展大繁荣，更好发挥文化引领风尚、教育人民、服务社会、推动发展的作用。

　　增强先进文化引导力。用社会主义核心价值体系引导社会思潮，倡导富强、民主、文明、和谐，倡导自由、平等、公正、法治，倡导爱国、敬业、诚信、友善。以“两个率先”目标凝聚社会力量，弘扬新时期江苏精神和南通精神，共建美好家园，同享文明成果。光大精神文明“南通现象”，鼓励凡人善举和见义勇为，推动形成良好社会风尚。加强诚信南通建设，健全文明城市创建长效机制，提升市民法制意识、道德素质和城市文明程度。

　　增强公共文化服务力。扩大公共文化产品覆盖面，推动文化服务向社区延伸、农村倾斜，创建一批省级公共文化服务体系示范区。加强公共文化设施建设，筹建市文化艺术中心，建成市图书馆新馆等工程，推进乡镇（街道）综合文化站和广播电视站标准化建设。增强文化创造活力，打造一批精品力作。繁荣发展哲学社会科学。加强历史文化保护，启动唐闸近代工业遗存申报世界文化遗产。

　　增强文化产业竞争力。落实加快文化产业发展的政策意见，文化产业增加值占GDP比重达到3.5%。促进文化与科技、旅游、金融深度融合，推动广播影视、民间工艺等传统产业特色发展，培育新型文化业态，创新文化产业发展模式，壮大一批文化企业。推动唐闸文化创意产业园争创国家示范园区，加快印刷工业园等文化产业园区建设，打造文化产业交易平台和公共服务平台，促进文化产业集聚发展。

　　（八）着力推进社会建设和管理，加快构建民生幸福城市。把人民对美好生活的向往作为工作方向，完善社会建设“十大体系”，办好10件40项惠民实事，增进民生幸福、和谐。

　　推进教育现代化建设。坚持办好人民满意的教育。推动各类教育协调发展。扩大公办幼儿园和省优质园覆盖面，创建省级学前教育改革发展示范区。深入实施素质教育，提升义务教育、高中教育优质均衡发展水平。加快职业教育和高等教育创新发展，增强服务地方发展能力。积极发展继续教育和特殊教育，完善终身教育体系。推动教育资源优化配置。优化学校布局，推进通大附中、通师新校区等项目建设，加强薄弱学校改造，促进教师合理流动，让更多孩子享受良好教育。推动教师队伍素质提升。加强队伍建设，提高师德水平和业务能力，培养一批名师、名校长。

　　增加城乡居民收入。落实各项富民措施，不断拓宽居民收入倍增空间，促进共同富裕。倡导辛勤劳动创造美好生活，突出就业创业富民，完善就业服务体系，新增就业9万人；实施第二轮“万人创业计划”，争创省级创业型城市。多渠道增加居民资产性、财产性收入，鼓励多种形式的股份合作，支持投资服务机构发展。继续增加转移性收入，推进新一轮脱贫工程。

　　完善社会保障体系。着眼于增强公平性、适应流动性、保证可持续性，健全覆盖城乡居民的社会保障体系。完善养老保障体系，确保城乡基本养老保险覆盖率达到98.5%，乡镇（街道）居家养老服务中心建成率达到70%，每千名老人拥有机构养老床位数达到27张。完善住房保障体系，市区住房保障覆盖面扩大到18%，安置拆迁户1.6万户。健全社会救助体系，提高城乡低保标准。健全残疾人社会保障和服务体系。加强关心下一代、红十字、慈善等工作。继续抓好“米袋子”、“菜篮子”工程。

　　提高人民健康水平。突出保基本、强基层、建机制，为群众提供安全有效方便价廉的公共卫生和基本医疗服务。健全全民医保体系，市区职工、城镇居民医保政策范围内住院费用补偿比例均提高2个百分点，加大重特大疾病救治救助力度。健全便民惠民医疗服务体系，巩固基本药物制度。完善人口计生服务体系，促进人口长期均衡发展。广泛开展全民健身活动，办好市十运会。

　　加强社会管理创新。健全体制机制，争创全国社会管理创新示范城市。强化基层管理和服务体系建设。着力增强城乡社区政务便民、文化体育、医疗卫生等服务功能，力争75%的城市社区、65%的农村社区达到省级和谐社区标准，城乡社区综合服务中心建设达标率超过95%。健全四级便民服务网络。引导社会组织健康有序发展。发挥工青妇和行业协会、商会等各类社团组织在社会管理中的作用。强化社会管理工作体系建设。完善民生诉求表达服务机制，打造“12345”政府服务热线“民心专列”品牌。全力推进社会管理强基工程，强化三级社会管理服务平台建设。更加注重源头治理，健全重大项目“稳评”、“环评”等机制。创新流动人口和特殊人群服务管理机制，建成人口基础数据库。强化公共安全体系建设。深化长安南通、法治南通建设，完善立体化社会治安防控体系，依法防范和惩治违法犯罪。抓好食品安全动态监测和长效监管。坚决遏制重特大安全事故。强化应急体系建设和防灾减灾工作。加强国防动员和后备力量建设。做好国家安全、民族、宗教和档案、保密等工作。

　　（九）着力推进环境质量提升，加快生态文明建设。推动绿色、循环、低碳发展，加快形成节约资源和保护环境的空间格局、产业结构和生产生活方式，全力建设国家生态市，努力把环境敏感期变成生态建设加速期。加强能源资源节约利用。大力发展循环经济，加快构建绿色经济产业链和资源循环利用链。坚持最严格的耕地保护和节约用地制度，优化国土空间开发格局，促进生产空间节约高效、生活空间宜居适度、生态空间山清水秀。严格水资源管理，推进节水型社会建设。加强生态建设和保护。深入推进“铁腕治污”，抓好重点区域、流域污染治理和重点行业标准化整治、化工园区生态化创建。提高环保基础设施建设和运营水平。下决心整治水环境，实施新一轮市区水环境综合整治三年行动计划，推进农村河道疏浚整治，实现水畅、水清、水美，营造水生态、水文化。加强海洋生态环境保护，建设国家海洋公园。打好大气环境整治硬仗，突出抓好秸秆禁烧等工作，确保PM2.5监测能力县（市）区全覆盖。加快新一轮绿色南通建设，确保市区新增绿地300公顷，全市林木覆盖率提高0.8个百分点。加强可持续发展制度建设。严格生态环境保护责任制，完善目标体系、考核办法和奖惩机制。健全环境信用评级等制度，开展绿色保险、排污权交易、环境区域补偿等试点。完善环保公众参与办法，营造生态文明建设良好氛围。

　　（十）着力推进服务型政府建设，加快转变政府职能。顺应“两个率先”新要求、人民群众新期待，以改革创新的精神，建设职能科学、结构优化、廉洁高效、人民满意的服务型政府。简政放权，促进提升效能。深化行政审批“三集中、三到位”工作，坚决取消一切不符合发展要求的审批事项，更大力度优化审批流程，最大限度简化政务、下放权力、降低收费，推动政府职能向创造良好发展环境、提供优质公共服务、维护社会公平正义转变。规范行为，促进依法行政。强化科学、民主、依法决策，健全决策机制、程序和决策问责、纠错制度。推进权力运行公开化、规范化，完善政务公开和各领域办事公开制度，促进阳光行政。健全行政执法责任制、过错责任追究制，推动严格规范公正文明执法。自觉接受人大法律监督和工作监督、政协民主监督，认真办好人大代表议案、政协建议案和人大代表建议、政协提案。重视公众监督、网络监督、舆论监督，加强审计监督。强化执行，促进高效运行。深化政府工作推进、协调、督查三大体系建设，强化目标化管理、项目化推进、责任化落实。完善县（市）区重点经济工作综合考核和政府系统“创新奖”评选办法，倡导敢于负责、勇于担当、善于突破、勤于落实。加强效能监察、执法监察和廉政监察，强化问责机制，严肃查办庸懒散等行政不作为、慢作为、乱作为行为。改进作风，促进勤政廉政。严格执行中央关于改进工作作风、密切联系群众的《八项规定》和省、市委《十项规定》，坚持求真务实、反对弄虚作假，坚持艰苦奋斗、反对奢侈浪费，多下基层调研，多为群众解难，大力精简会议活动，大幅减少文件简报，把时间和精力用在促发展、抓落实上。深入推进政府系统惩防体系和制度建设，坚决反对腐败，促进干部清正、政府清廉、政治清明。加强公共资源交易中心、综合电子监察中心功能建设，强化对重要部门、关键环节的权力运行监管，防控廉政风险。加强财政专项资金使用绩效评估。切实强化公务员能力和作风建设，努力打造一支想干事、能干事、让群众满意的公务员队伍。

　　各位代表，新形势、新任务对我们提出了新的更高要求。让我们紧密团结在以习近平同志为核心的党中央周围，在省委、省政府和市委的坚强领导下，同心同德、真抓实干，锐意创新、开拓进取，为把南通建设得更加美好而努力奋斗！
